# Improving the Immunogenicity of the *Mycobacterium bovis* BCG Vaccine by Non-Genetic Bacterial Surface Decoration Using the Avidin-Biotin System

**DOI:** 10.1371/journal.pone.0145833

**Published:** 2015-12-30

**Authors:** Ting-Yu Angela Liao, Alice Lau, Sunil Joseph, Vesa Hytönen, Zakaria Hmama

**Affiliations:** 1 Division of Infectious Diseases, Department of Medicine and Vancouver Costal Health Research Institute, University of British Columbia, Vancouver, BC, Canada; 2 Institute of Biomedical Technology, University of Tampere, Tampere, Finland; University of Delhi, INDIA

## Abstract

Current strategies to improve the current BCG vaccine attempt to over-express genes encoding specific *M*. *tuberculosis* (Mtb) antigens and/or regulators of antigen presentation function, which indeed have the potential to reshape BCG in many ways. However, these approaches often face serious difficulties, in particular the efficiency and stability of gene expression via nucleic acid complementation and safety concerns associated with the introduction of exogenous DNA. As an alternative, we developed a novel non-genetic approach for rapid and efficient display of exogenous proteins on bacterial cell surface. The technology involves expression of proteins of interest in fusion with a mutant version of monomeric avidin that has the feature of reversible binding to biotin. Fusion proteins are then used to decorate the surface of biotinylated BCG. Surface coating of BCG with recombinant proteins was highly reproducible and stable. It also resisted to the freeze-drying shock routinely used in manufacturing conventional BCG. Modifications of BCG surface did not affect its growth in culture media neither its survival within the host cell. Macrophages phagocytized coated BCG bacteria, which efficiently delivered their surface cargo of avidin fusion proteins to MHC class I and class II antigen presentation compartments. Thereafter, chimeric proteins corresponding to a surrogate antigen derived from ovalbumin and the Mtb specific ESAT6 antigen were generated and tested for immunogenicity in vaccinated mice. We found that BCG displaying ovalbumin antigen induces an immune response with a magnitude similar to that induced by BCG genetically expressing the same surrogate antigen. We also found that BCG decorated with Mtb specific antigen ESAT6 successfully induces the expansion of specific T cell responses. This novel technology, therefore, represents a practical and effective alternative to DNA-based gene expression for upgrading the current BCG vaccine.

## Introduction

Despite major efforts to reduce the global tuberculosis (TB) burden, recent World Health Organization (WHO) report indicates that there were an estimated 8.6 million new TB cases in 2012 with 1.3 million TB deaths worldwide including countries having advanced health systems (WHO global tuberculosis report, 2013), which places TB as the second leading cause of death from infectious diseases after AIDS. Moreover, the spread of drug-resistant *Mycobacterium tuberculosis* (Mtb) strains over the past decade presents an unprecedented challenge to TB control. Even more troubling is the recent emergence of new strains of totally drug-resistant TB strains, for which there are no treatment options [1]. On the other hand, the increasing rates of co-infection with HIV [2] is also largely contributing to the spread of TB [3,4], thus adding more complexity to TB control globally. Given that an untreated TB patient can infect up to 15 contacts in a year [5], in the near future, the speed of spread of TB will largely surpass treatment success rates and one would worry that TB will again become an incurable disease

It is generally accepted that protective vaccination is the best and most cost-effective option to prevent the spread of infectious diseases. We do have a vaccine, Bacillus Calmette–Guérin (BCG), developed in early 1900's. It is a live attenuated vaccine that is given annually to about 100 million newborns worldwide with proven safety and efficacy records in the protection against severe TB meningitis and other disseminated TB [6]. Unfortunately BCG fails to protect pulmonary TB in adults, the most prevalent form of the disease [7,8]. Therefore, upgrading BCG to improve its efficacy while maintaining its level of safety is highly recommended [9] and if successful, it would be transformational for public health with huge benefits across society, especially in developing countries.

Current strategies to improve BCG are based on introducing recombinant genetic material to over-express BCG antigens that are immunogenic but not expressed sufficiently during infection [10] or Mtb-specific antigens that are absent in BCG [11]. Other approaches aim to complement BCG with gene encoding co-factors that potentiate antigen presentation function [12,13]. Genetic engineering holds great potential for developing novel and efficient BCG-derived TB vaccines. However, this great promise is facing many barriers to the routine use of recombinant BCG strains for large-scale vaccinations. Major limitations include the uncertain level of safety and low efficiency of expression vectors [14,15]. This raises the question of whether an alternate approach of vaccine improvement can be achieved by complementing BCG with exogenous proteins of interest, rather than nucleic acids.

We believe that reversible display of proteins of interest (antigens or immunostimulants) on bacterial cell surface would safely permit broad manipulations of BCG to achieve maximal improvement of its efficacy. To this end, we made use of the high-affinity interaction of streptavidin with biotin, which offers the possibility to attach avidin fusion proteins on the surface of bacteria that have been modified with biotin. However, for this approach to serve as an alternative to gene transfer methods, it should allow for long-term display of proteins, and the process of bacterial surface biotinylation and decoration with exogenous proteins should not compromise either the phenotype of the bacterium or the properties of the proteins displayed at its surface. We tested this approach using a fusion protein consisting of an ovalbumin (OVA) antigen domain and a new version of low affinity monomeric avidin (K_d_ = 10^−7^ M) to ensure for slow release of the antigen from the surface of biotinylated BCG once ingested by antigen presenting cells. The choice of OVA antigen is to develop a proof of concept study using widely available and easy to use research tools to study immune response to this popular surrogate antigen [16,17]. Our data demonstrate that BCG cell surface can be easily modified with biotin for rapid display of exogenous avidin fusion proteins without detectable change in bacterial phenotype and that bacteria decorated with surrogate OVA antigen are efficiently ingested by antigen presenting cells *in vitro* consistent with the capacity to induce a specific T cell response *in vivo*. Similar observations were made with BCG surface decorated with the Mtb specific antigen ESAT6. Therefore, this novel method for rapid display of recombinant proteins on BCG cell surface provides an efficient alternative for gene transfer approaches with broad applications in vaccine development and in cellular mycobacteriology research.

## Results

### Generation of plasmids expressing monomeric avidin fusion proteins

We previously designed a mutant version of chicken avidin (N54A and W110K) in order to convert homotetrameric avidin, which binds four molecules of biotin, into a monomeric form (mAvidin) [18]. The affinity for biotin binding of mAvidin decreased from *K*_*d*_ ∼10^−15^ M of the wild-type tetramer to *K*_*d*_ ∼10^−7^ M resulting in reversible avidin binding to biotin [18]. In this study we designed a more elaborated version of mAvidin by adding an additional mutation (N17I) in order to prevent N-linked glycosylation, which would minimize undesired non-specific binding of glycosylated avidin to cells and tissues. These new features of mAvidin perfectly suit our purpose of transient and specific display of individual chimeric molecules on the surface of biotinylated BCG without inducing their aggregation.

**Fig 1:**
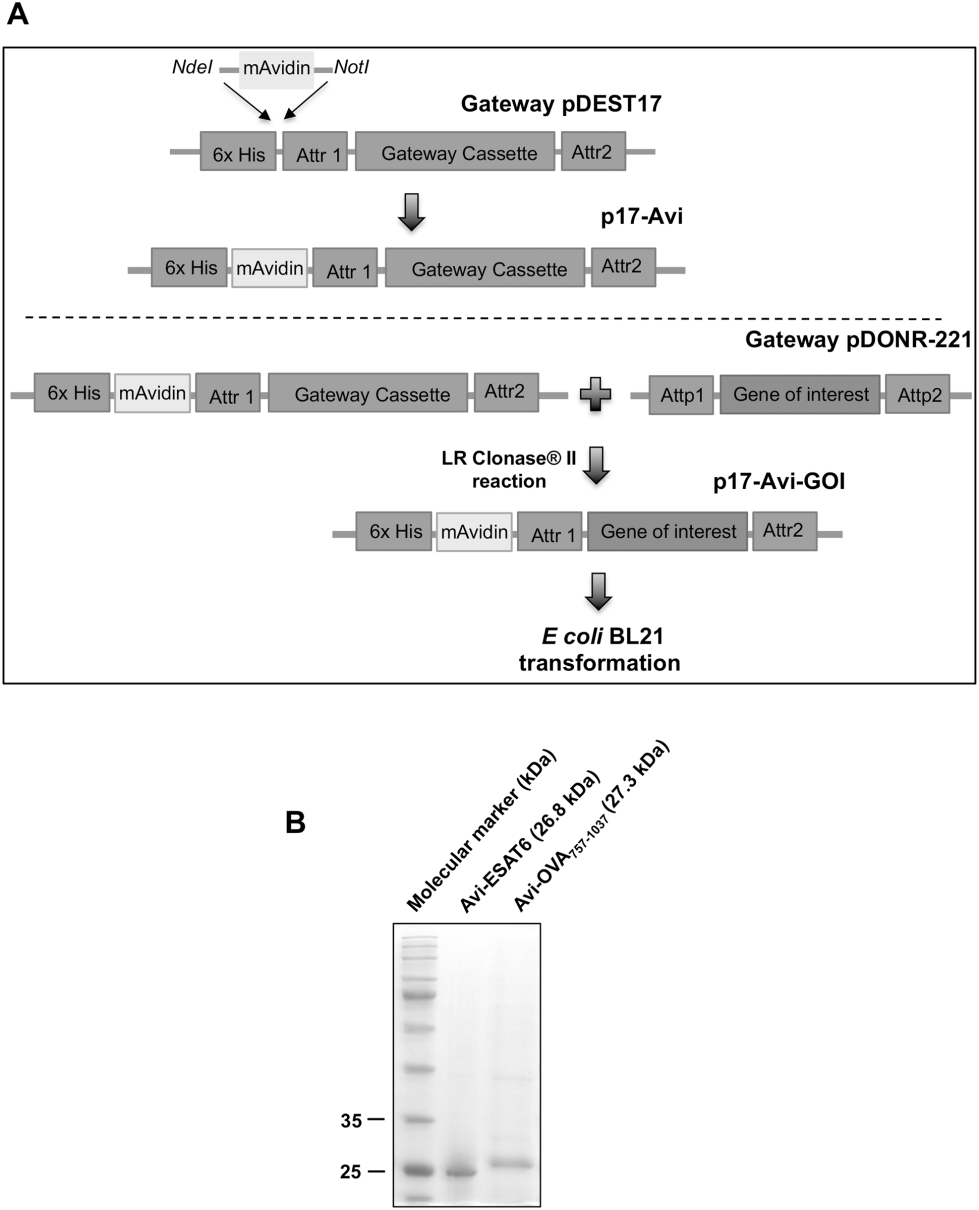
Construction of a recombination cloning plasmid for the production of avidin fusion proteins. (A) Gene for mAvidin was synthesized with restriction sites NdeI and NotI and subcloned into pDEST17 between the 6x histidine and the gateway cassette to generate p17-Avi plasmid. OVA peptide_323-339_ and ESAT6 DNA sequences terminated with *att*B sites were cloned into pDONR221 via BP Clonase reaction. Thereafter, genes of interest were subcloned into p17-Avi by mean of one step LR Clonase reaction. (B) *E*. *coli* BL21 was transformed with p17-Avi encoding ESAT6 and OVA peptide_323-339_. Recombinant Avi-proteins were purified from inclusion bodies and subjected to 12% SDS-PAGE gel and EZ blue staining to analyze the quality of fusion protein preparations. Expected sizes for Avi-ESAT6 and Avi-OVA_757-1037_ are 26.8 and 27.3 kDa respectively.

To produce mAvidin fusion proteins, we generated a novel expression plasmid compatible with the Gateway methodology for rapid and efficient, restriction enzyme-free, recombination cloning of gene of interests in frame with mAvidin (Fig 1). DNA sequence encoding mutant mAvidin was subcloned into pDEST17 plasmid as described in the Material and Method section and nucleotide sequence analysis confirmed the expected insertion of mAvidin between the "CTC" and "GAA" (133-134bp, i.e., between the 6-histidine encoding sequence and the pDEST17 recombination site *Attr1*). The resulting destination plasmid was named “p17-Avi”.

To test the expression efficiency of p17-Avi, DNA sequence corresponding to OVA_757-1037_ peptide, was constructed by PCR amplification using gene-specific primers flanked with Attb adapters (Table 1) and cloned into Gateway pDONR-221 (Invitrogen) as recommended by the manufacturer. We also cloned in ESAT6 which is a major antigen secreted by Mtb [19]. Genes of interest were then transferred into p17-Avi by mean of LR Clonase reaction (Fig 1A). Fusion proteins were expressed in *E*. *coli* then purified and refolded as described in the Material and Method section. SDS-PAGE analyses (Fig 1B) showed single bands of Avi-OVA_757-1037_ and Avi-ESAT6 proteins, which migrate towards the expected molecular weight position of mAvidin fusion proteins. mAvidin-OVA_757-1037_ (Avi-OVA) is used as a surrogate antigen to test the efficiency of biotin-avidin based decoration of BCG with exogenous antigens.

**Table 1:** List of primers used in this study.

Primer	DNA target	Sequence
Attb1-OVA	Puc57-BCGOVA	GGGGACAAGTTTGTACAAAAAAGCAGGCTTCCTTGAGCAGCTTGAGAGTAT
Attb2-OVA	Puc57-BCGOVA	GGGGACCACTTTGTACAAGAAAGCTGGGTGTTACCCTACCACCTCTCTGC
Attb1-ESAT6	TB genomic DNA	GGGGACAAGTTTGTACAAAAAAGCAGGCTTCATGACAGAGCAGCAGTGGAA
Attb2-ESAT6	TB genomic DNA	GGGGACCACTTTGTACAAGAAAGCTGGGTGTCATGCGAACATCCCAGTGA

Attb1 and Attb2 sequences are underlined

### Biotin binds efficiently to BCG cell surface

**Fig 2:**
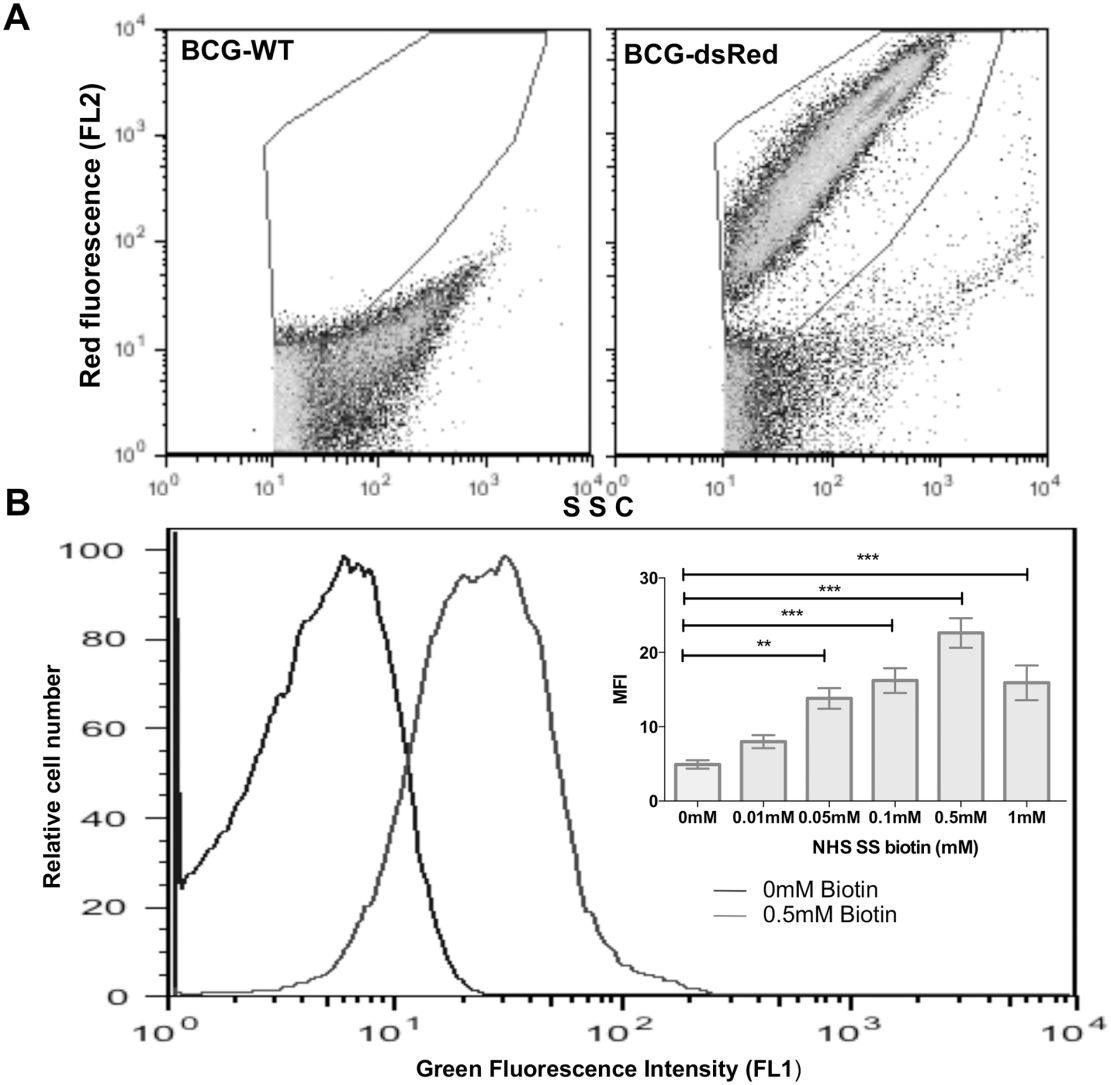
Efficiency of BCG biotinylation. (A) SSC and FL2 parameters and red fluorescent BCG were used to allow for proper gating of true bacteria during FACS analyses. (B) dsRed-BCG was biotinylated with various concentration of NHS-SS-biotin for 30 min at room temperature, then labeled with FITC-streptavidin and analyzed by FACS. Results are presented as histograms of green fluorescence intensity and the insert graph represents the mean±SEM of mean fluorescence intensity (MFI) deducted from 3 independent experiments. *P<0.05; **P<0.01; ***P<0.001.

To examine the efficiency of BCG surface biotinylation we labeled bacteria expressing red fluorescence (dsRed-BCG) with various concentrations of Sulfo-NHS-SS-Biotin (0-1mM) and measured the relative abundance of bound biotin by staining with FITC-conjugated streptavidin and FACS analysis. The use of fluorescent bacteria allows for discriminating true bacteria from small-size contaminating particles in most FACS running buffers, which coincide with dots corresponding to BCG particles in SSC vs FSC displays (Fig 2A). Fluorescent histograms deducted from FACS analyses showed a direct relationship between Sulfo-NHS-SS-Biotin concentration and the level of biotin detected on the bacterial surface (Fig 2B). A total shift of fluorescence histogram (i.e. ~100% positive events) was observed in bacterial samples labeled with 0.5mM biotin and this concentration was used to generate biotinylated (Biot)-BCG throughout this study.

### Biotinylation of BCG does not affect its phenotype

**Fig 3:**
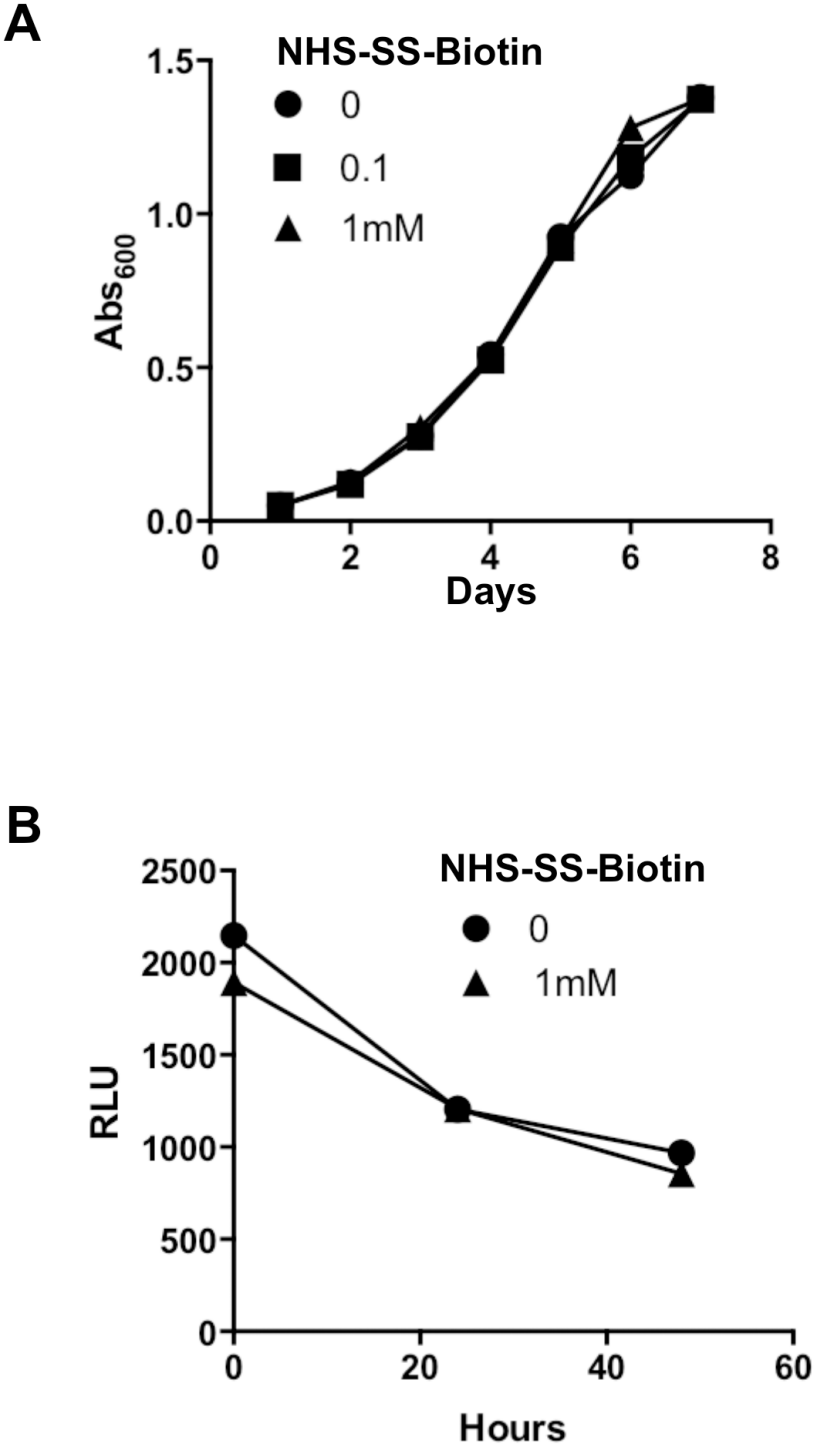
Biotinylation of BCG surface does not affect its growth or its survival in the macrophages. (A) Biot-BCG and control unlabeled wild-type BCG were grown in complete 7H9 media and replication was monitored over an 8-day period. The results are expressed as growth curves, i.,e., absorbance at 600nm as function of time. (B) Macrophages were infected with Biot-BCG-Luc or control unlabeled BCG-Luc for the indicated time periods, and then cell lysates were prepared and assayed for bioluminescence to detect viable bacteria. Results are expressed as relative light units (RLU). Data shown are representative of three independent experiments.

We next examined the effect of bacterial surface modification, as result of biotinylation, on the growth kinetics in culture media. The result obtained showed that Biot-BCG displays a growth profile similar to that of control untreated bacteria, over a 8-day period (Fig 3A). On the other hand, we took advantage of our BCG strain expressing luciferase (BCG-Luc) [20] to examine Biot-BCG survival in the macrophage. Luminescence signals recorded (Fig 3B) showed that biotinylated and control bacteria display similar profiles of gradual viability decrease within the macrophage. Taking together, these data clearly demonstrate that surface modification with biotin is compatible with bacterial growth and more importantly, does not increase BCG persistence in the macrophage, which would be interpreted as a conversion into a virulent bacterium.

### Surface decoration of BCG is efficient, stable and do not affect phagocytosis and intracellular trafficking inside macrophages

**Fig 4:**
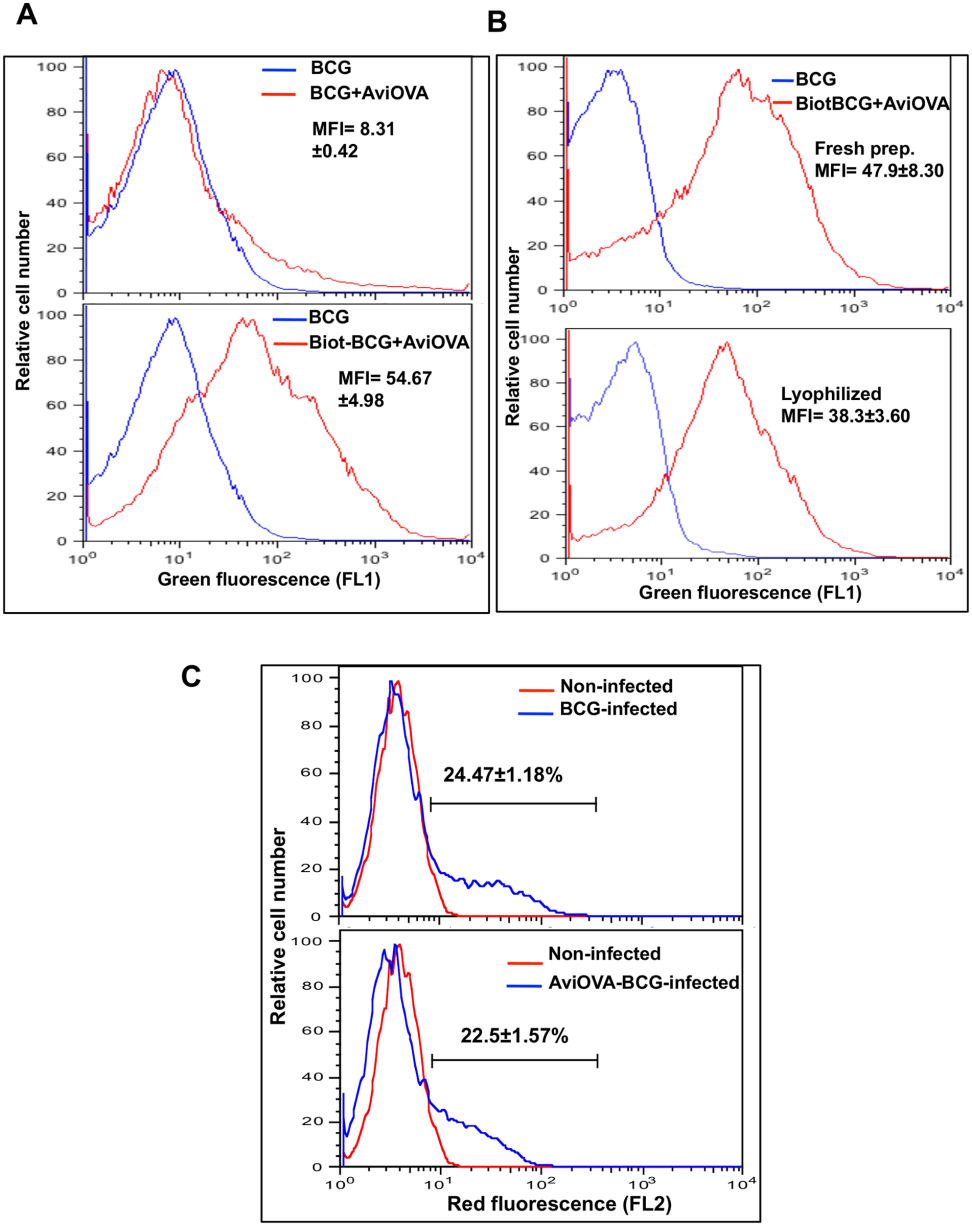
mAvidin-OVA binding to Biot-BCG, its stability and delivery inside macrophages. (A) Biot-dsRed-BCG and control non-biotinylated dsRed-BCG were mixed with Avi-OVA at room temperature for 1 h and the extent of OVA binding was evaluated by surface staining with rabbit anti-avidin Ab and FITC- goat anti-rabbit IgG (FL1). Sample were washed and analyzed by FACS. Results are presented as histogram of green fluorescence intensity with mean± SEM of mean fluorescence intensity (MFI) of Biot-BCG-AviOVA from three independent experiments. (B) Lyophilized Biot-BCG coated with Avi-OVA and freshly made Biot-BCG with Avi-OVA were labeled and analyzed by FACS as described in A. Data shown are representative of three independent experiments. Mean of MFI±SEM for biotinylated BCG coated with Avi-OVA are shown. (C) RAW macrophages were infected with OVA-dsRed-BCG or dsRed-BCG for 24 h at 37°C. Samples were washed, treated with trypsin and fixed and analyzed by FACS. Results are expressed as red fluorescence histogram, which reflect the extent of phagocytosis. Values indicated average percentage ±SEM of cells ingesting BCG. Results shown are representative of three independent experiments.

Initial experiments examined the extent and success of Biot-BCG surface decoration with exogenous proteins. Biot-dsRed-BCG and control non-biotinylated bacteria were exposed to OVA antigen peptide (Avi-OVA, 10 μg/ml) for 1 h at room temperature. Bacteria were then washed and bound OVA was detected with rabbit anti-avidin Ab. FACS analyses show a minimal binding of OVA peptide to non-biotinylated bacteria (MFI = 8.31 ± 0.42, Fig 4A top panel). In contrast, significant levels of OVA peptide were detected on the surface of Biot-BCG as reflected by total shift of fluorescence histograms corresponding to Avi-OVA coated Biot-BCG (MFI = 54.67±4.98) relative to control uncoated bacteria (MFI = 5.92±0.22, Fig 4A lower panel). These data demonstrated the efficiency and specificity of mAvidin fusion protein binding to the surface of biotinylated bacteria.

Since conventional BCG vaccines are formulated as dried powders, we examined whether the freeze-drying process affects the stability of upgraded BCG with the biotin-avidin approach described above. To do so, aliquots of biotinylated bacteria coated with Avi-OVA were lyophilized as described in the Material and Method section then compared to freshly made bacterial preparations for surface display of Avi-OVA. FACS data (Fig 4B) show that lyophilized bacteria, reconstituted one month later, retained substantial levels of avidin-OVA on its surface (MFI = 38.3±3.6) compared to levels detected on fresh Avi-OVA-BCG preparations (MFI = 47.9±8.3). This test demonstrates that surface decoration of BCG with exogenous protein is stable and reproducible.

Next, we used RAW264.7 cells and red-fluorescent bacteria to verify whether surface modification of BCG with biotin and Avi-OVA peptide bound to it interferes with its entry into host macrophages. Phagocytosis assays were performed as described in the Material and Method section. BCG uptake by macrophages was quantified by FACS and fluorescence histograms shown in Fig 4C indicate that BCG surface modification has minor inhibitory effect on its uptake and ingestion by the macrophage (22.5±1.57% red fluorescent cells), compared to the level of uptake of control unlabeled BCG wild type (24.47±1.18% red fluorescent cells).

**Fig 5:**
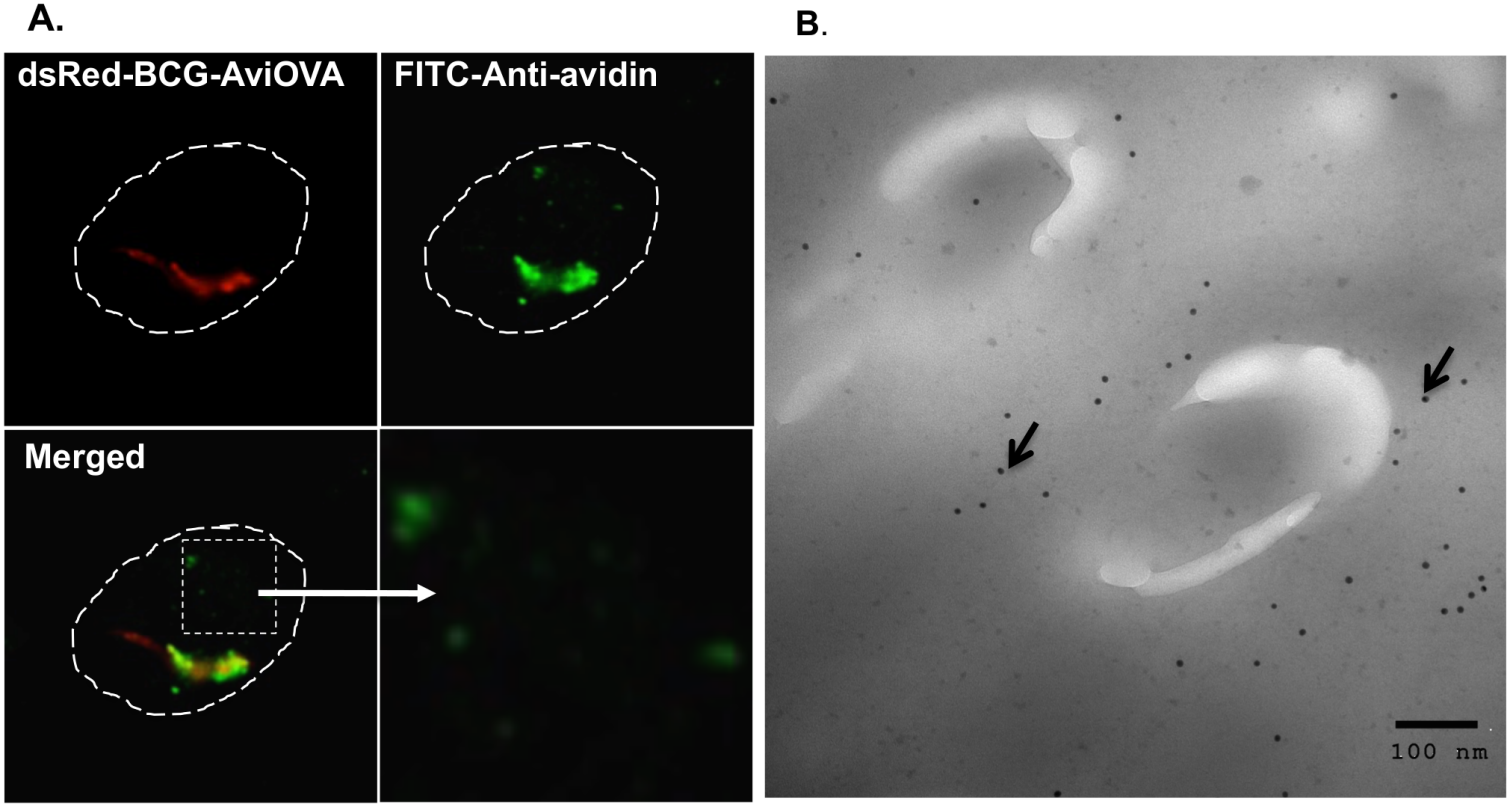
mAvidin-OVA detaches from BCG surface and crosses the phagosomal membrane toward the cytosol. (A) Adherent RAW cells on cover slips were infected with OVA-decorated dsRed-BCG for 24 h at 37°C and then fixed/permeabilized and stained with rabbit anti-avidin Ab and FITC- goat anti-rabbit IgG. Sample were mounted on microscope slides and analyzed by digital confocal microscopy. Red signal indicates the position of BCG and green signal reflects the localization of Avi-OVA. Dotted line indicates the macrophage cell boundary and the bottom right panel is a 4 X magnification of the insert shown in left panel. (B) Thin sections of BCG-AviOVA infected RAW cells were fixed with paraformaldehyde and incubated sequentially with anti-avidin Ab and gold-conjugated goat anti-rabbit IgG to visualize Avi-OVA. EM grids were examined with a electron microscope images and magnification of 12,000x were shown. The arrowheads indicate Avi-OVA dissociated from BCG surface and exported beyond the phagosome membrane. The images shown are representatives of two independent experiments.

To examine the fate of OVA-decorated BCG ingested by the macrophage, adherent RAW cells on cover slips were infected as described above and subjected to intracellular staining with rabbit anti-avidin Ab and FITC-conjugated anti-rabbit IgG. Thereafter, cover slips were mounted on microscope slides and examined by digital confocal microscopy as described [21]. Results obtained (Fig 5A) show a substantial and diffused green fluorescent signal far distant from ingested BCG particles. Thereafter we performed immunogold staining and EM analyses, which clearly demonstrated that OVA antigen detaches from BCG surface and effectively crosses the phagosomal membrane toward the cytosol (Fig 5B).

Taking together, these data indicate that BCG surface decoration with exogenous protein does not affect interaction of bacterial ligands with specific macrophage surface receptors involved in phagocytosis and that once it enters into macrophages, BCG surface decorated with antigens is able to export its surface cargo towards other cell compartments.

### Avidin-fusion antigens co-localize with MHC molecules

**Fig 6:**
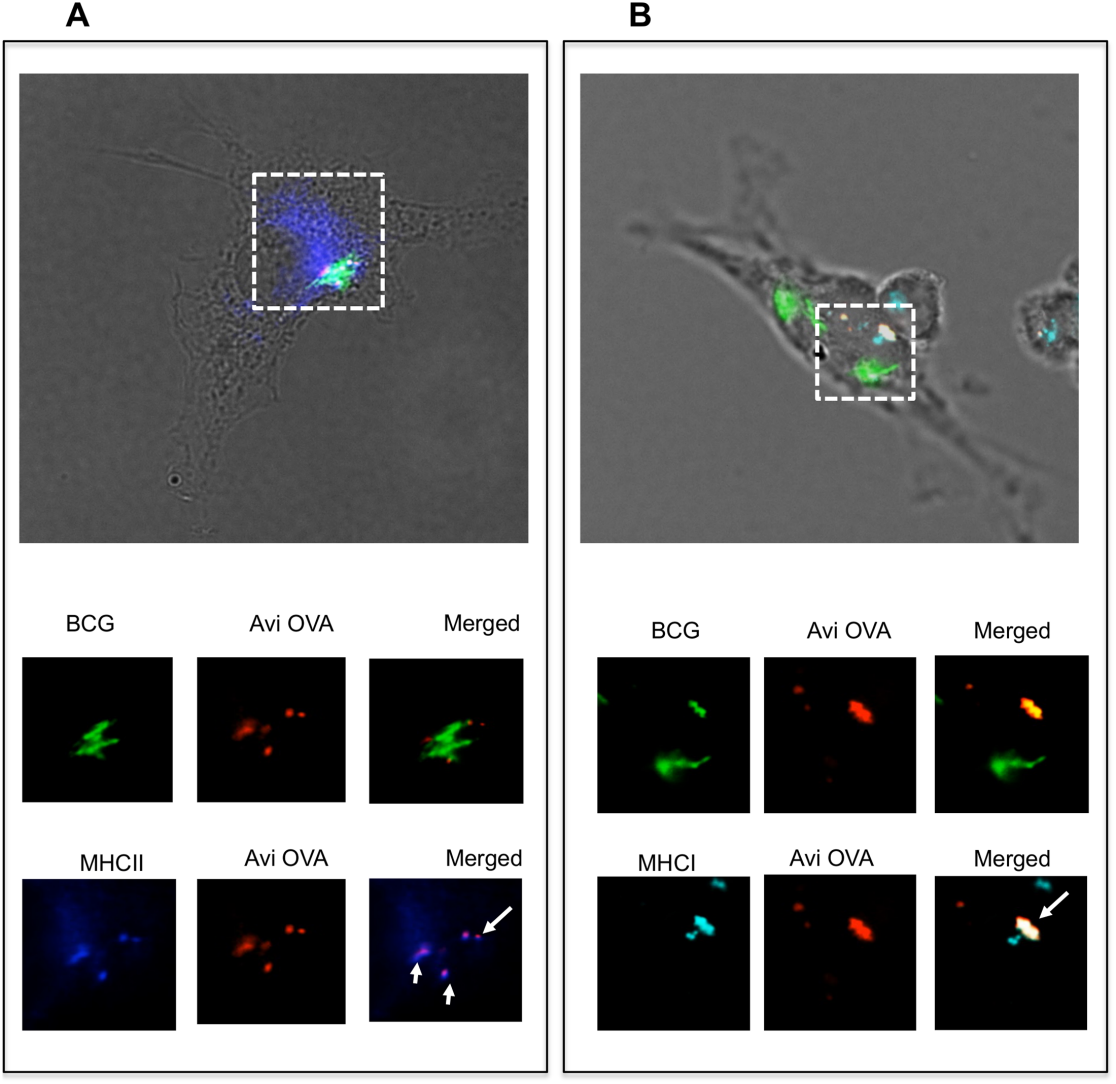
Avidin-fusion antigen co-localizes with MHC class II and class I molecules. Adherent BMA cells were infected with OVA decorated GFP-BCG for 4 h and then simulated with IFN-γ for 24 h. Cells were then fixed/permeabilized and stained with rabbit anti-avidin Ab, Alexa 594 anti-rabbit IgG and either Alexa 647 rat anti-mouse I-A (A) or Alexa 647 rat anti mouse H-2k^b^ (B). Samples were mounted on microscope slides and analyzed by digital confocal microscopy. Green signal indicates the position of BCG-GFP and red signal reflects the localization of Avi-OVA. Blue signal indicates the position of MHC class II or class I molecules. Dotted line indicates area of interest. Arrowheads indicated Avi-OVA colocalization with MHC molecules. Images shown are representatives of two independent experiments.

BMA3.1A7, a mouse macrophage cell line commonly used to study antigen presentation [22] was infected with GFP-BCG surface decorated with Avi-OVA and subjected to intracellular staining for OVA peptide and I-A. Confocal images obtained show that Avi-OVA co-localize with I-A (Fig 6A) molecules suggesting that avidin fusion antigen molecules dissociated from the surface of biotinylated bacteria and translocated to compartments specialized for antigen processing and loading into MHC class II molecules. On the other hand, intracellular staining for H-2k^b^ showed abundant co-localization of class I molecules and OVA peptide within BCG phagosomes (Fig 6B), suggestive of potential presentation of OVA antigen to CD8^+^ T cells by the macrophage, consistent with previous studies showing that phagosomes are competent organelles for antigen cross-presentation [23].

These data clearly demonstrate that once ingested by the macrophage, modified bacteria are capable of delivering their surface antigen cargo to the antigen presentation machinery.

### Biot-BCG surface decorated with surrogate OVA antigen is fully immunogenic

Results presented above clearly demonstrate the effectiveness and robustness of avidin/biotin-mediated protein surface display developed in this study. In particular, the findings of intracellular trafficking of exogenous antigens dissociated from BCG surface suggest that they join compartments specialized in antigen processing and presentation to T cells. Since our ultimate objective is to improve BCG vaccine efficacy via surface display of immunodominant antigens we judged essential to examine whether BCG decorated with surrogate antigen OVA is able to induce a specific immune response *in vivo*. To do so, B6 mice were immunized with wild-type BCG or BCG-Avi-OVA or PBS alone (control). Animals were euthanized 20 days later to perform *ex-vivo* experiments. We also examined whether immunogenicity of antigen surface decorated BCG is comparable to that of BCG genetically engineered to express a similar antigen. Thus, mice were immunized with BCG transformed with plasmid expressing OVA_757-1035_ in fusion with the 19 kDa surface lipoprotein (BCG-p19-OVA) and BCG-p19-Avi-OVA, which correspond to BCG expressing p19 alone and surface decorated with Avi-OVA. The amount of OVA present on BCG-p19-OVA and BCG surface decorated with Avi-OVA are comparable (S3 Fig). To determine the frequency of OVA-specific CD4^+^ T cells, splenocytes were labeled with PE-conjugated I-A^b^-OVA_323-339_ tetramers (FL2) and AF647 CD4 (FL4) then analyzed by flow cytometry as described in the Material and Methods section, and in the **Supplemental Figure** (S4 Fig).

**Fig 7:**
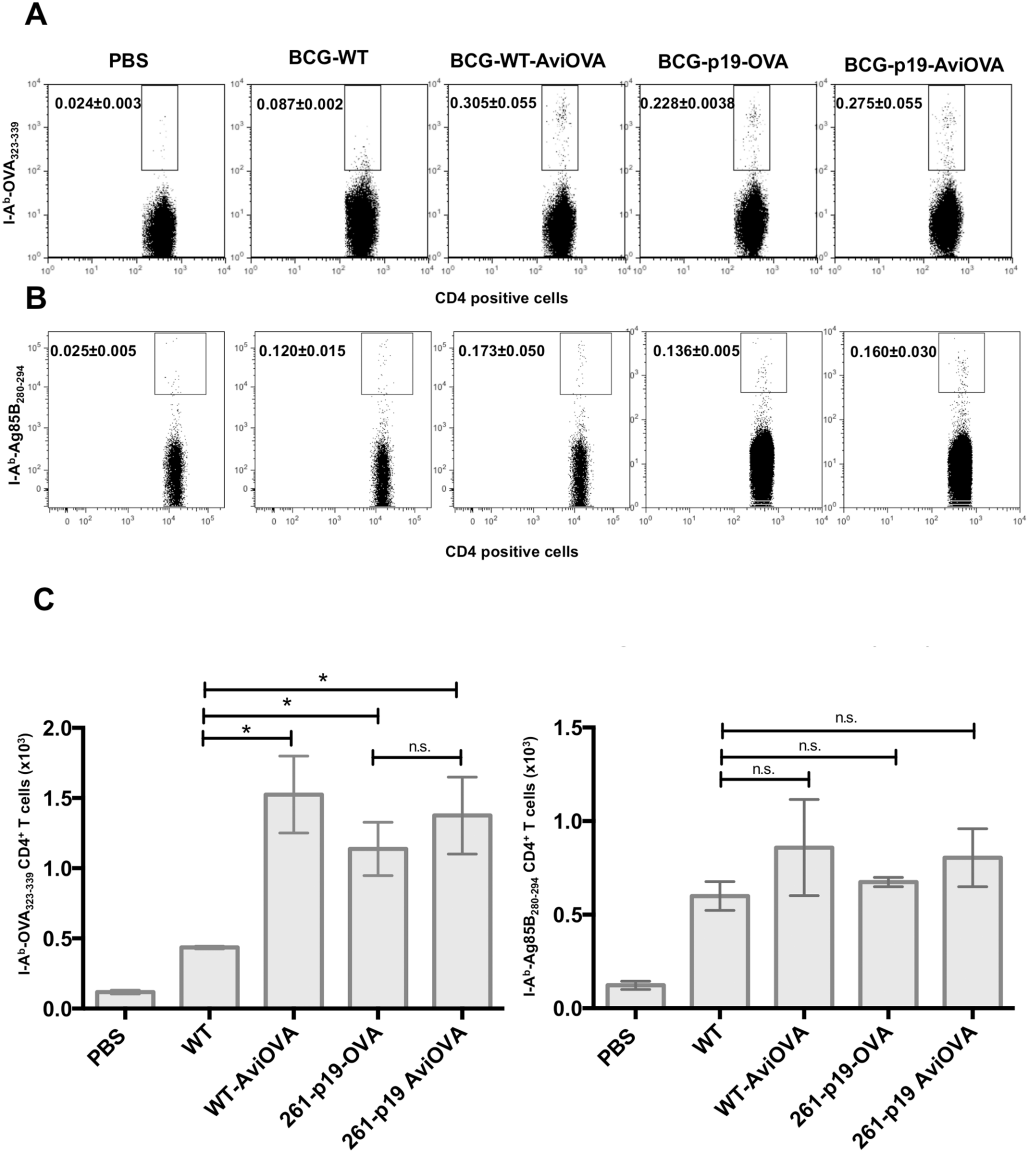
*In vivo* CD4^+^ T cell response to OVA-decorated BCG. C57BL/6 mice were injected subcutaneously with BCG surface decorated with OVA, unmodified BCG wild-type, PBS (control), BCG genetically transformed to express OVA (BCG-p19-OVA) or transformed with control plasmid and surface decorated with Avi-OVA (BCG-p19-AviOVA). After a 20-day period, splenocytes were prepared from the spleens of euthanized animals and stained with PE-conjugated I-A^b^-OVA_323-339_ tetramers (A) or PE-conjugated I-A^b^-Ag85B_280-294_ tetramer (B) followed by AF647-CD4 Ab and 7-AAD. Samples were then analyzed by FACS. Results are expressed as two-parameter dot plots that show the average frequencies± SEM of tetramer positive events in the CD4^+^ population from two animals/group. Data shown are representative of three independent experiments. The data in graphs (C) are expressed as mean value± SEM of the absolute number (in a total of 500,000 events) of OVA Tetramer specific CD4^+^ cells in (left graph) or Ag85B Tetramer specific CD4^+^ T cells (right graph) from two animals/group and three independent experiments. *P<0.05; **P<0.01; ***P<0.001.

FL4 *vs* FL2 dot plots show a significantly larger proportion of tetramer positive events in the panel corresponding to animals immunized with wild-type BCG-Avi-OVA (0.305±0.055%) compared to almost no positive events in those corresponding to animals receiving BCG wild-type or PBS (0.087±0.002% and 0.024±0.003% respectively) (Fig 7A and 7C left graph, which indicate absolute numbers of tetramer positive cells). On the other hand, OVA-specific CD4^+^ T cell responses to BCG-p19-Avi-OVA (tetramer positive events = 0.275±0.055%) are similar to those induced by BCG-p19-OVA (0.228±0.038%) and not significantly different from those corresponding to control wild-type BCG-Avi-OVA (Fig 7A and 7C, left graph). These findings indicate that a significant expansion of OVA specific CD4^+^ T cells occurred in vaccinated animals and that the degree of immunogenicity of OVA surface coated BCG is comparable to that of BCG genetically expressing similar OVA epitope.

Besides I-A^b^-OVA_323-339_ tetramer staining, splenocyte samples were also subjected to staining with PE-conjugated I-A^b^-Ag85B_280-294_ tetramers to detect Ag85B-specific CD4^+^ T cells in the treatment groups listed above. Results obtained indicate that there are no significant differences in the frequencies (Fig 7B) and absolute numbers (Fig 7C right graph) of Ag85B-specific CD4^+^ T cells between untreated wild type BCG and surface modified BCG preparations via the avidin-biotin system or plasmid transformation. These findings clearly demonstrate that surface biotinylation and addition of exogenous antigens to BCG surface do not impact the immune response to its native antigens.

**Fig 8:**
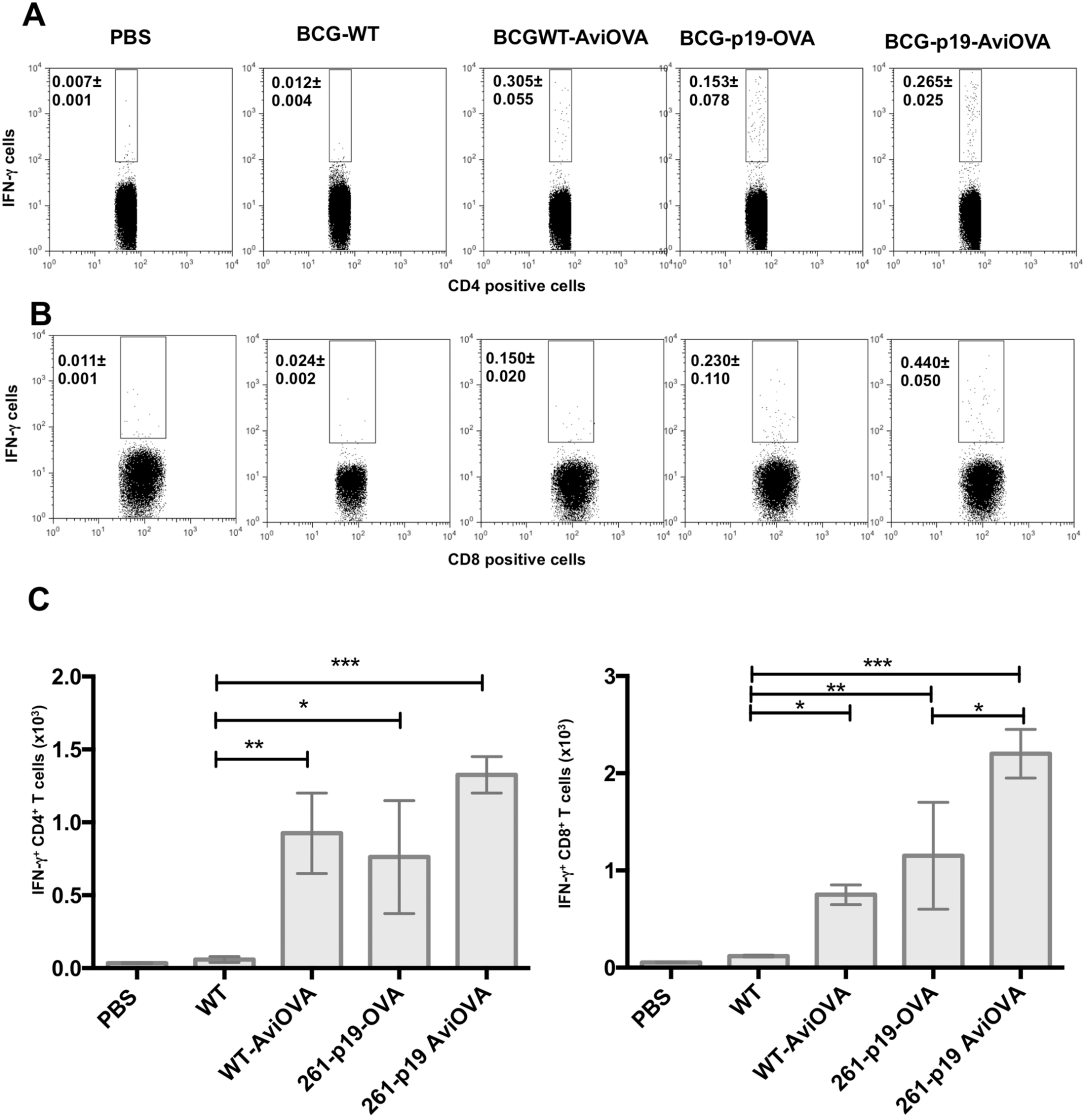
Frequencies of T cells releasing cytokines in response to Avi-OVA coated BCG-immunization. Splenocytes from immunized mice with PBS, BCG Wild-type, BCG WT surface decorated with Avi-OVA, BCG-p19-OVA and BCG-p19-AviOVA were stimulated with recombinant OVA protein for 16 hours followed by a 5 h-period treatment with Brefeldin A. Cells were then washed and stained first with PE-Cy7 anti-CD4 (A) or PE anti- CD8 Ab (B) then AF647 anti-IFN-γ Ab. Cells were then washed and analyzed by FACS. Results are expressed as two-parameter dot plots to show the average frequencies± SEM of IFN-γ producing cell subsets in CD4^+^ and CD8^+^ populations from two animals/group. Data shown are representative of three independent experiments. The data in graphs (C) are expressed as the mean of absolute number± SEM of IFN-γ releasing CD4^+^ T cells (left graph) or IFN-γ releasing CD8^+^ T cells (right graph) from two animals/group and three independent experiments. *P<0.05; **P<0.01; ***P<0.001.

IFN-γ is known to play an important role in the protective response against intracellular pathogens, including Mycobacteria [24]. Therefore, to further visualize specific T cell responses to OVA-decorated BCG, we performed ICS experiments to determine the frequencies of OVA-specific T cells releasing cytokines in immunized animals. Splenocytes were pulsed *ex vivo* with recombinant OVA and subjected to CD4 (FL3) immunostaining along with IFN-γ (FL4) as described in the Material and Methods section. Data deducted from FACS analyses show similar level of OVA-specific IFN-γ releasing CD4^+^ T cells in animals immunized with BCG WT-Avi-OVA, BCG-p19-OVA and BCG-p19-Avi-OVA. In fact, the average frequencies of IFN-γ positive and CD4 positive events (0.305±0.055%, 0.153±0.078% and 0.265±0.025% respectively) are significantly higher than those obtained in response to wild-type BCG (0.012±0.004%) (Fig 8A). These data are consistent with the absolute numbers of IFN-γ positive and CD4 positive events (Fig 8C left graph).

Given that CD8^+^ T cell responses efficiently contribute to TB immunity [25], we extended our ICS experiments to include analyses of cytokine releasing CD8^+^ T cell in response to OVA antigen by double immunostaining of splenocytes for CD8 and IFN-γ. Frequency (Fig 8B) and absolute number (Fig 8C right graph) data reveal a significant expansion of IFN-γ producing CD8^+^ T cells in splenocytes isolated from mice immunized with Avi-OVA-coated wild-type BCG (0.150±0.020%) relative to data deducted from mice inoculated with untreated BCG (0.024±0.002%). More importantly, CD8^+^ T cell response in splenocytes isolated from BCG-p19-OVA immunized animals (0.230±0.110%) is significantly lower than those observed in splenocytes from BCG-p19-Avi-OVA inoculated mice (0.440±0.050%). These differences are consistent with the absolute numbers of cytokine releasing CD8^+^ T cell shown in Fig 8C right graph.

Taking together, these data demonstrated that BCG transformation with antigen-encoding nucleic acids could be effectively replaced by biotin/avidin mediated antigen surface display methodology.

### Evaluation of the immunogenicity of biot-BCG decorated with ESAT6

**Fig 9:**
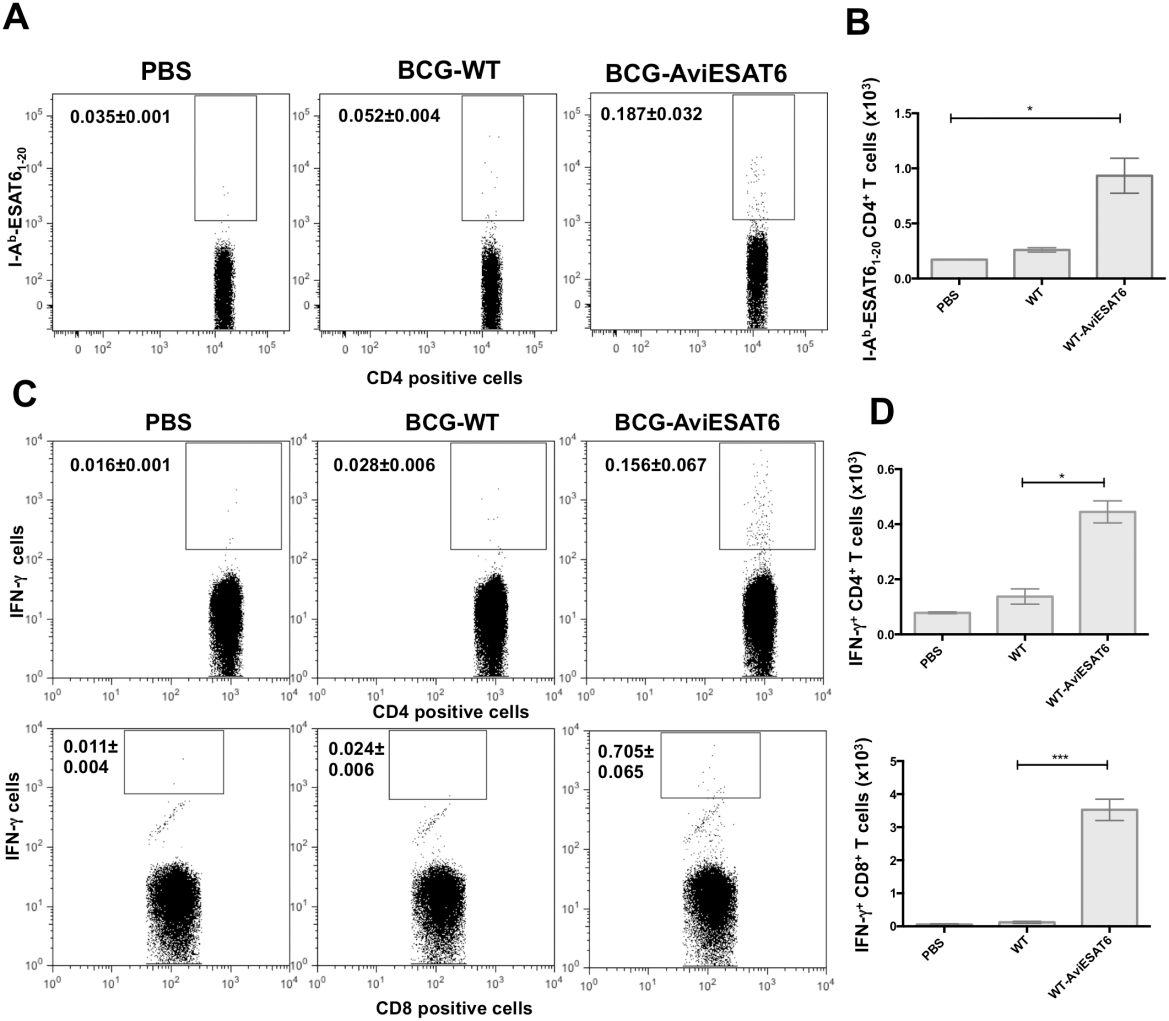
*In vivo* CD4^+^ T cell response to ESAT6-decorated BCG and frequencies of T cells releasing cytokines in response to ESAT6. C57BL/6 mice were immunized with PBS, BCG WT alone or BCG WT surface decorated with Avi-ESAT6 as described in Fig 7. (A) Splenocytes from immunized animals were stained with PE-conjugated I-A^b^-ESAT6_1-20_ tetramers followed by AF647-CD4 Ab and 7-AAD. Samples were then analyzed by FACS. Results are expressed as two-parameter dot plots that show the average frequencies± SEM of tetramer positive events in the CD4^+^ population from two animals/group. Data shown are representative of three independent experiments. The data in graphs (B) are expressed as mean values of the absolute number± SEM of IFN-γ releasing CD4^+^ T cells (left graph) or IFN-γ releasing CD8^+^T cells (right graph) from two animals/group and three independent experiments. (C) Splenocytes from immunized animals were stimulated with recombinant ESAT6 protein then stained and analyzed by FACS as described in Fig 8. Results are expressed as two-parameter dot plots to show the average frequencies± SEM of IFN-γ producing cell subsets in CD4^+^ (upper panel) and CD8^+^ (lower panel) populations from two animals/group. Data shown are representative of three independent experiments. (D) Each column in graph represents the mean of absolute number± SEM of IFN-γ releasing CD4^+^ (Left graph) or CD8^+^T cells (right graph) from two animals/group and three independent experiments. *P<0.05; **P<0.01; ***P<0.001.

Experiments using the surrogate antigen OVA clearly demonstrated the efficiency of surface decorated BCG to induce specific immune response *in vivo*. To validate this approach for Mtb specific proteins, we examined mouse immune response to BCG surface decorated with the early secreted Mtb antigen ESAT6, which is a major immunodominant antigen not expressed in BCG [11] and have been previously shown to induce protective T cell response against virulent Mtb [26]. Data from tetramer staining with PE-conjugated I-A^b^-ESAT6_1-20_ tetramer (Fig 9A) show that splenocytes from BCG-Avi-ESAT6 immunized mice contained a substantial ESAT6 specific CD4^+^ T cell subset (0.187±0.032% tetramer positive events) relative to the background value (0.052±0.004%) obtained from splenocytes from wild-type BCG immunized mice. This is also shown in the absolute number of ESAT6 specific CD4^+^ T cell numbers (Fig 9B). On the other hand, ICS showed significant expansion of IFN-γ producing CD4^+^ T cells in response to ESAT6 antigen in splenocytes isolated from BCG-Avi-ESAT6 (0.156±0.067%) compared to BCG WT (0.028±0.006%) vaccinated animals (Fig 9C top panel and Fig 9D top graph). Furthermore, the frequencies of IFN-γ producing CD8^+^ T cells in BCG-Avi-ESAT6 immunized mice were significantly greater than in mice immunized with wild-type BCG (Fig 9C bottom panel and Fig 9D bottom graph). Mice immunized with BCG coated with Avi-ESAT6 were able to induce 0.705±0.065% ESAT6 specific IFN-γ producing CD8^+^ T cells compared to 0.024±0.006% in BCG WT immunized mice. Taken together, these data showed that BCG surface coated with Mtb specific protein, ESAT6, was able to successfully induce specific ESAT6 immune response *in vivo* and improve immunogenicity of BCG.

These additional data strongly support the approach of upgrading BCG by mean of surface display of protective Mtb antigens.

## Discussion

Today, an effective TB vaccine is more urgently needed than ever in order to reverse the current worrisome burden of global TB epidemic. In this regard, many TB investigators suggested building on the success of BCG to protect children in order to develop novel protective TB vaccines. BCG has so far been given to more than 3 billion people with rare serious adverse outcomes [27] and therefore genetics reshaping is being considered as a reasonable approach to further improve its efficacy. This strategy is currently being used extensively for expression of exogenous antigen proteins or proteins with defined immunological properties in BCG [15,28], but only few recombinant BCG vaccines are currently being seriously considered for clinical trials [15]. Although the use of conventional gene transfer via plasmid-mediated transformation holds great promise for the developing TB vaccines, a series of difficulties remain to be overcome in order to speed up the development of alternative versions of recombinant BCG strains.

Replicative plasmids have been successfully used to express high levels of heterologous genes in BCG [29]. However, their stability during bacterial culture wanes with time [30], which is a drawback to mass production of recombinant vaccines. On the other hand, the use of integrative vectors, although more stable than replicative plasmids [14], leads to limited levels of protein expression. Thus, optimization of BCG as a vehicle for live recombinant vaccines requires development of alternative strategies for efficient antigen expression.

We reasoned that a method that allows for rapid and reversible display of exogenous proteins on the cell surface might offer a viable alternative to gene transfer approaches. In this regard, we report here an effective method based on the biotin-avidin system to achieve stable and efficient display of exogenous proteins to either add specific antigens or specific functional properties to bacterial cell surface expected to efficiently improve BCG immunogenicity. The procedure is performed in a short period of time (~ 2 h) without detectable change of bacterial phenotype. Use of this method results in the immediate presence of functional proteins on the cell surface without the time lag (3 to 6 months) required for transformation and selection of positive clones and their characterization. Another advantage of this method is the possibility to adjust the amount of chimeric proteins on the cell surface by varying the concentration of recombinant avidin fusion protein, allowing simultaneous display of several proteins on the bacterial cell surface and thus maximizing the effectiveness of vaccine candidates.

Avidin binding to biotin is the strongest non-covalent interaction known in nature (K_d_ ∼ 10^−15^ M) [31], and this tight binding is one of the most general tools for biological research [32]. In recent years, it has been extensively developed and approved for many therapeutic applications, including cancer treatments [33,34]. In an attempt to adapt the properties of avidin for our purpose, i.e., a form of avidin that binds reversibly to biotin, we applied site-directed mutagenesis (N54A and W110K) to wild-type avidin, which converted homotetrameric avidin into a monomeric form (mAvidin). mAvidin has a significantly reduced affinity to biotin (K_d_ ∼ 10^−7^ M) and thus binds reversibly to biotin [18]. In the present study we mutated the glycosylation site of avidin in order to produce a non-glycosylated form of avidin and showed that this mutation did not generate deleterious effects on the biotin-binding properties. This novel form of avidin was used to develop a novel plasmid (p17-Avi) for rapid expression of protein of interest using the Gateway recombination cloning method. Of note, p17-Avi would also be useful for rapid expression of many human or mouse mAvidin chimera molecules from ORFs (cytokines, apoptosis inducers or transcription factors) already subcloned in Entry vectors compatible with recombination cloning (http://orf.invitrogen.com).

It is well known that naturally occurring anti-avidin antibodies are common in humans and laboratory animals [35]. Thus one may think these antibodies would hamper the use of avidin for therapeutic purpose. However, this is not the case since detailed clinical studies demonstrated that safety and efficacy of avidin are not significantly affected by its immunogenicity [36]. In this regards, it is important to note that conversion of tetrameric avidin to a monomer is associated with a significant decrease of its immunogenicity [37]. Low affinity monomeric avidin has the advantage to circumvent two additional and major drawbacks. First, monomeric avidin prevents aggregation, which would result in large bacterial clumps useless for vaccinal purposes. Second, reversible binding allows for transient surface display of molecules of interest on BCG surface. Thus, once ingested by the host cell, cellular mechanisms of the phagocytic cells are able to detach from the surface of modified bacteria their cargo and deliver it intracellularly. Finally, non-glycosylated monoavidin could also be an attractive system for production of large quantities of avidin chimera molecules in eukaryotic systems like yeast or transgenic plants [38,39].

Our *in vitro* studies clearly showed that surface decoration of BCG with monoavidin fusion proteins did not affect its uptake and internalization by host macrophages. Thereafter, the use of advanced FACS-based exploratory assays, which include MHC tetramer technology and ICS allowed us to objectively evaluate the T-cell axis and Th1 cytokine, particularly IFN-γ detection, in animals immunized with modified BCG preparations. We clearly demonstrated that surface decorated bacteria are as immunogenic as BCG genetically engineered to express similar antigens. Furthermore, the expansion of specific T cells secreting cytokines in response to BCG surface decorated with the well documented ESAT-6 antigen indicated that this novel and timely technology represents a great tool to evaluate rapidly and accurately the protective efficacy of many secreted specific Mtb proteins thought to induce protective TB immunity.

Many studies, including from our laboratory, showed that BCG interferes with important macrophage functions associated with phagosome maturation arrest [40], including decreased antigen presentation [41]. Therefore the release of avidin fusion protein from the surface of ingested bacteria and its trafficking within the cytosol open up many other possibilities to further improve BCG. Indeed, one possibility would be to label BCG surface with a combination of antigens of interest and constitutively active effectors known to accelerate phagosome biogenesis.

**Fig 10:**
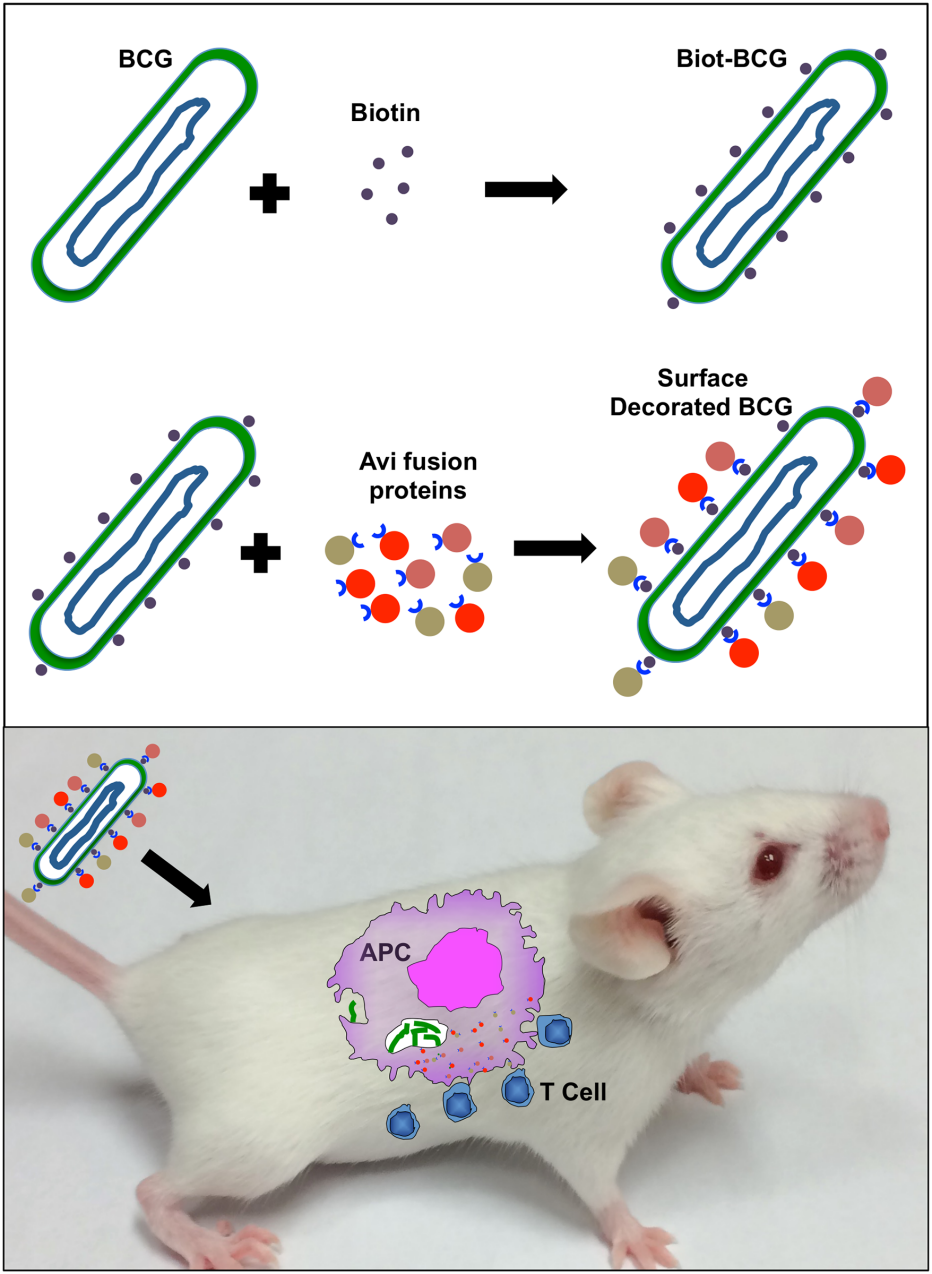
Schematic summary of BCG surface decoration approach. BCG is first surface biotinylated with hydrosoluble biotin then exposed to mAvidin chimeric proteins for reversible surface decoration with antigens. Modified BCG is then inoculated into animals where it is expected to deliver antigens to induce specific T cell responses. Mouse picture was taken by one of the contributor to this work.

In conclusion, our study provides strong data to support proof-of-concept for a novel strategy aimed at optimizing BCG for vaccinal purposes (Fig 10). It is based on rapid, reproducible and reversible surface decoration with one or multiple proteins of interest via binding of avidin chimeric proteins to biotinylated bacteria capable of delivering their cargo inside antigen presenting cells *in vivo*. Overall, this study revealed the enormous potential of the avidin-biotin system in TB vaccine development and hopefully would also give new insights for other successful therapeutic applications.

## Material and Methods

### Reagents and chemicals

Endotoxin-free RPMI 1640 and miscellaneous culture reagents were purchased from StemCell Technologies (Vancouver, BC, Canada). Fetal calf serum (FCS) was purchased from Gibco Laboratories (Burlington, ON, Canada). Protease inhibitor mixture and PMSF were purchased from Sigma-Aldrich (St. Louis, MO). Sulfo-NHS SS biotin, FITC-conjugated streptavidin and NDSB256 were purchased from Sigma-Aldrich. Phycoerythrin (PE)-conjugated I-A^b^-OVA_323-339_ tetramer and PE-conjugated I-A^b^-ESAT6_1-20_ tetramer were purchased from MBL International (Woburn, MA). Phycoerythrin (PE)- conjugated I-A^b^-Ag85B_280-294_ tetramer was acquired from NIH. 7-AAD was purchased from BD Pharmingen.

### Antibodies

Alexa Fluor (AF) 647 conjugated rat anti-mouse CD4, AF647 rat anti-mouse CD8, AF647 rat anti-mouse IFN-γ, AF647 rat anti-mouse I-A/I-E, PeCy7 rat anti-mouse CD4, PE rat anti-mouse CD8 Ab, AF 647 rat anti mouse H-2k^b^ were purchased from BD Bioscience (Mississauga, ON, Canada). Rabbit polyclonal anti-avidin Ab was described previously [18]. Ultra small gold-conjugated goat anti-rabbit IgG was purchased from Electron Microscopy Sciences (Hatfield, PA).

### Mycobcterial strains

Wild-type BCG Pasteur was obtained form Dr. Richard Stokes (Department of Microbiology and Immunology, University of British Columbia). BCG-p19, a recombinant BCG strain over expressing mycobacterial surface antigen 19-KDa lipoprotein (LpqH, Rv3763), was generated using our integrative pJAK1.A plasmid (selection marker, kanamycin [20]) encoding the full-length LpqH gene as described [20]. BCG expressing ovalbumin (OVA) surrogate antigen (BCG-p19-OVA) was engineered by transformation with pJAK1.A encoding fusion of LpqH with OVA polypeptide (amino acids 757–1035), which covers both H-2K^b^-restricted (SIINFEKL) and I-A^b^-restricted (KISQAVHAAHAEINEAG) epitopes [42–45]. The resulting plasmid was named pJAK1.A-19-OVA. dsRed-BCG (red fluorescent), Luc-BCG and GFP-BCG (green fluorescent) were previously described [20]. Wild-type BCG and its derivative strains were grown in Middlebrook 7H9 broth (BD Diagnostic Systems, Mississauga, ON, Canada) supplemented with 10% (v/v) OADC (oleic acid, albumin and dextrose solution; BD Diagnostic Systems) and 0.05% (v/v) Tween 80 (Sigma-Aldrich, St. Louis, MO) at 37°C on a shaker platform at 50 rpm. Bacterial aliquots from exponentially growing culture and depleted from aggregates (3–5 x 10^8^ CFU/ml) were stored at minus 80°C.

### Bioluminescence detection of survival of bacteria in macrophages

RAW264.7 cells (ATCC, Manassas, VA, USA), a mouse macrophage cell line commonly used in mycobacterial studies were infected with Biot-BCG-Luc or untreated BCG-Luc (control) and incubated at 37°C over a 48 h-period. Cell monolayers were washed and then lysed with 0.025% SDS to release ingested bacteria. Bioluminescence production, indicative of bacterial viability, was measured using Bright-Glo^™^ Luciferase assay system (Promega, Madison, WI) and a Turner Biosystem luminometer (Model TD-20/20, Promega) as described [46].

### Phagocytosis assays

RAW264.7 cells were exposed to dsRed-BCG decorated with Avi-OVA or untreated dsRed-BCG (control) at MOI 20:1. After an incubation period of 24 h at 37°C, cells were extensively washed and partially attached and non-ingested bacteria were removed by trypsin treatment as described [47]. Samples were then fixed and analyzed using a BD FACSCalibur flow cytometer (BD Biosciences) and histograms of red fluorescence values, which reflect the extent of phagocytosis, were constructed.

### Expression plasmid for monomeric Avidin fusion protein

Destination vector for protein expression in fusion with monomeric avidin (mAvidin), by mean of recombination cloning, was prepared as follow: DNA sequence encoding triple mutant avidin (N17I, N54A and W110K) protein (S1 Fig) was synthesized with 3’ and 5’ restriction sites *NdeI* and *NotI* respectively and subcloned into pDEST17 plasmid (Invitrogen, Life Technologies, Burlington, ON, Canada) to obtain a novel Gateway cloning plasmid, p17-Avi, compatible with the one-step and restriction enzyme-free recombination Gateway cloning methodology.

### Preparation of mAvidin fusion antigens

Initially we produced mAvidin in fusion with OVA surrogate antigen (I-A^b^- and H-2K^b^-restricted epitopes). DNA sequence corresponding to OVA polypeptide_757-1035_ (S2 Fig) was PCR-amplified with 3’ and 5’ primers flanked with *att*B1 and *att*B2 sequences respectively (Table 1), using pUC57-OVA plasmid (GenScript, Piscataway, NJ) as template. The resulting PCR product was used for directional cloning into pDONR-221 using BP clonase (Invitrogen) to obtain pDONR-OVA. Thereafter, OVA DNA ORF was transferred into p17-Avi through site-specific *in vitro* recombination using the LR clonase (Invitrogen). The resulting plasmid, p17-Avi-OVA was transformed into *E*. *coli* BL21 and protein expression was induced with IPTG (0.1 M) for 3 h at 37°C. Bacteria were lysed and 6x-histidine-Avi-OVA protein was purified from the inclusion bodies fraction using Ni-NTA columns (Qiagen, Toronto, Ontario, Canada). Eluted protein was refolded by gradual dilution (1:10) and rapid vortexing in Tris buffer (pH 7.5) containing 1mM DTT, 200mM NDSB256, 0.5mM T80, 500mM arginine and 200mM NaCl for 30 min at room temperature. Refolded protein was subjected to desalting and buffer exchange into PBS using Pierce protein concentrators (Thermo Fisher Scientific, Rockford, IL). Aggregates were removed by 30 min centrifugation (3,500 *xg*) at 4°C and aliquots of soluble protein were stored at minus 20°C. Similar strategy was used to express and refold mAvidin fusion with Mtb antigens ESAT6 (Rv3875).

### BCG biotinylation and surface decoration with mAvidin fusion antigens

BCG was washed three times with cold PBS plus 0.1% Tween80 (PBS-T), then resuspended in PBS alone and labeled with various concentration of Sulfo-NHS SS biotin 30 min at room temperature. Labeled bacteria were washed twice with cold PBS-T to remove free biotin and re-suspended in PBS-T. To evaluate biotinylation efficiency, bacteria were labeled with streptavidin-FITC (1:100 dilution) for 20 min at room temperature, washed twice with PBS-T and subjected to FACS analysis.

Biotinylated bacteria were incubated with mAvidin fusion proteins (10μg/ml final in PBS-T) for 1 h at room temperature. Bacteria were then washed twice with PBS-T and stained with rabbit anti-avidin Ab (1:100 dilution) for 20 min at room temperature then FITC conjugated goat anti-rabbit IgG Ab in the same conditions. Bacteria were washed twice with PBS-T and analyzed by FACS to evaluate the extent of surface decoration.

### Lyophilization of bacteria

Bacteria were biotinylated and coated with Avi-OVA protein as described above. Bacterial aliquots (10^8^) were washed and resuspended in 0.5ml lyophilization media (25% Sauton medium, 75% H2O and 1.5% Na-glutamate),filled in Wheaton boroscilicate glass vials and transferred into a -80°C freezer over-night. Filled and frozen glass vials were lyophilized for 24 hours using a Novalyphe NL 500 freeze dryer (Savant Instruments, Holbrook, NY, USA). Dried samples were then stored at 4°C and reconstituted in PBS when needed.

### Fluorescence microscopy

Coverslips were mounted on microscope slides and examined by digital confocal microscopy as described [48].

### Immunoelectron microscopy

Immunogold staining was conducted at the EM Facility of the University of British Columbia (Vancouver, BC, Canada). In brief, BCG-infected macrophages were fixed with 4% paraformaldehyde, embedded in 4% low melting point agarose and dehydrated in ethanol. Samples were then transferred to LR White resin. After polymerization at 50°C, 60 nm sections were cut with a Leica EM UC6 microtome (Leica Microsystems, Switzerland) and collected on nickel grids. Samples were labeled with avidin antibodies then gold-conjugated F(ab')_2_ of ultra-small goat-anti-rabbit IgG. Sections were then washed in distilled water, stained in 2% glutaraldehyde, washed again, air dried and examined with Tecnai G2 200kV electron microscope (FEI Company, Hillsboro, OR).

### Animal immunization and organ processing

Female C57BL/6 mice (I-A^b^, H-2K^b^, 5–6 week old) were obtained from Charles River Laboratories (Sherbrooke, QC, Canada) and were housed under specific pathogen-free conditions in the animal biosafety level II facilities of the Jack Bell Research Centre (Vancouver, BC, Canada).

Mice were immunized subcutaneously in the scruff of the neck with wild-type BCG or its derivatives (1 x 10^6^ bacteria in 100 μl endotoxin-free PBS). Control mice received 100 μl PBS alone. Mice were euthanized 20 days post-immunization by carbon dioxide inhalation followed by cervical dislocation and spleens were excised and transferred into RPMI. Spleens were mashed through 70-μm Falcon cell strainer with a 5-ml syringe plunger and washed with 5 ml RPMI. Single cell suspensions were then centrifuged (800 x *g*, 3 min) and RBC was depleted by EasyStep mouse biotin positive selection kit (StemCell, Vancouver BC Canada) with biotin-Ter119/Erythroid cells Ab. Cells were centrifuged, resuspended in 10 ml complete RPMI (10% FCS, 1% L-glutamine, 1% penicillin, 1%/ streptomycin and 50 μM 2-ME) and counted.

### I-A^b^ tetramer staining

To determine the frequency of antigen-specific CD4^+^ T cells, splenocytes from control and immunized mice (~20 x 10^6^ cells) were stained in binding buffer (PBS with 2% FCS and 0.1% NaN_3_) with PE-conjugated I-A^b^-OVA_323-339_ tetramers (1/12.5 dilution) or PE-conjugated I-A^b^-ESAT6_1-20_ tetramer (1/25 dilution) for 1 h at 37°C followed by the addition of AF647 CD4 Ab (1:25) and 7-AAD (to detect dead cells) for 20 min at room temperature. Samples were then analyzed by flow cytometry. Gating method for quantification of tetramer positive lymphocytes is described in the **Supplemental Figure** (S4 Fig): Total splenocytes were defined by the SSC/FSC dot blot (region R1), live cells (R2) by exclusion of the 7-AAD positive events and CD4 or CD8 subsets (R3) by gating AF647 positive events respectively. A total of 500,000 events were acquired in R3 region to determine the frequencies of tetramer positive events, shown in the R4 region.

### Intracellular cytokine staining (ICS)

Approximately 1 × 10^7^ splenocytes were cultured in 4 ml of complete RPMI medium in six-well plates with or without recombinant OVA (10 μg/ml) or ESAT6 (10ug/ml) for 16 h. Cells were then treated with Brefeldin A (BD Pharmingen) for an additional 5 h, washed with PBS and subjected to ICS as follow: cell samples were first stained with PE-CD8 or PE-Cy7-CD4 Ab, fixed, and permeabilized using Cytofix/Cytoperm kit (BD Pharmingen) according to the manufacturer’s instructions. Cells were then washed and labeled with AF647-conjugated IFN-γ Ab for 20 min at room temperature. Cells were washed and subjected to FACS analysis as described above.

### Statistical analyses

Data are expressed as mean ± SEM. To analyze statistical differences between two groups, unpaired student *t*-test was employed. To analyze differences of more than two groups, one-way ANOVA and Tukey’s post-test for multiple comparisons was performed. P<0.05 was considered significant and represented by the symbol *. *P<0.05; **P<0.01; ***P<0.001.

### Ethics Statement

All animals were maintained in accordance with protocols approved by the Animal Care and Use Committees at the University of British Columbia. Experiments were approved by the Animal Care and Usage Committees and performed according to the Canadian Council on Animal Care Guidelines. The animal assurance welfare number is A11-0247.

## Supporting Information

S1 Fig DNA and protein sequence of OVA derived antigen peptide. **A.** DNA sequence shows partial sequence of the OVA gene encoding for a 92 amino acid polypeptide (757–1035) that covers both MHC class I-restricted (SIINFEKL) and MHC class II-restricted (KISQAVHAAHAEINEAG) OVA epitopes. The two epitopes are underlined. **B.** Protein sequence of OVA showing both MHC class I-restricted (SIINFEKL) and MHC class II-restricted (KISQAVHAAHAEINEAG) OVA epitopes (underlined).
(TIF)

S2 Fig Mutant monomeric avidin. **A.** DNA sequence showing the three mutations (N17I, N54A & W110K) introduced in wild-type avidin to obtain a monomeric avidin. **B.** Protein sequence of triple mutant avidin.
(TIF)

S3 Fig OVA levels in BCG genetically expressing OVA *vs* BCG surface decorated with Avi-OVA. BCG 261-p19-OVA and BCG 261-p19 decorated with Avi-OVA were fixed and stained with mouse anti-OVA Ab and the level of OVA on cell surface was revealed with FITC-anti-mouse IgG. Samples were then analyzed by FACS. Results are presented as MFI indexes, which correspond to the Ratios: MFIs deducted from OVA expressing BCG 261-p19/ MFI corresponding to control BCG-p19 alone.
(TIF)

S4 Fig Flow cytometry-gating strategy and dead cell exclusion. Splenocytes acquired on FACSCalibur flow cytometer were gated on a SSC/FSC (region R1). Live cell (7-AAD negative) were gated in region R2. Total CD4^+^ (or CD8^+^) T cells were gated in region R3 to determine frequencies of tetramer positive events (R4).
(TIF)
