# Supplementary figures and images for: Improving the Immunogenicity of the Mycobacterium bovis BCG Vaccine by Non-Genetic Bacterial Surface Decoration Using the Avidin-Biotin System
Source: PLoS One. 2015 Dec 30;10(12):e0145833. doi: 10.1371/journal.pone.0145833 (PMC4696857; doi:10.1371/journal.pone.0145833)

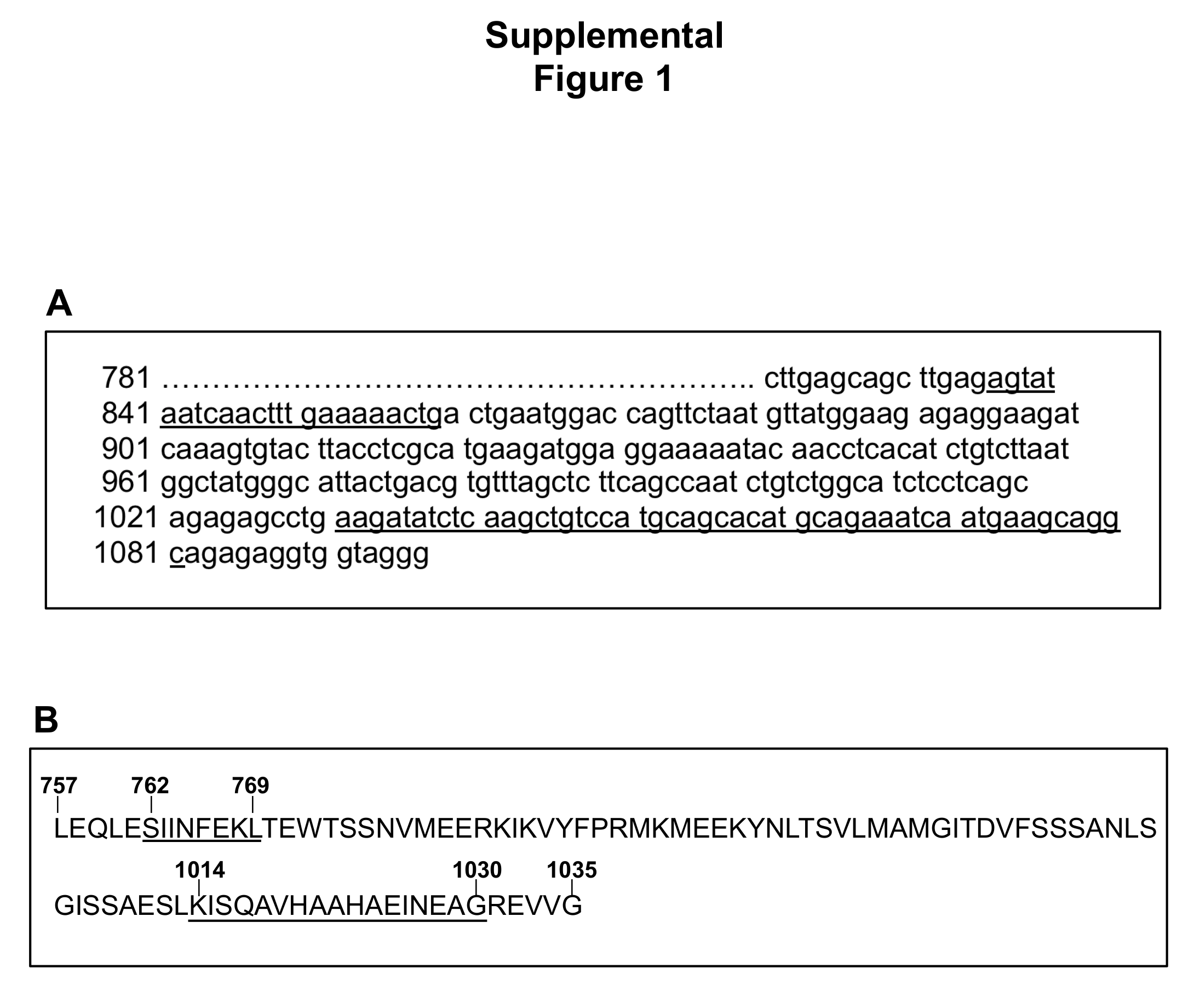

Supplement: S1 Fig — A. DNA sequence shows partial sequence of the OVA gene encoding for a 92 amino acid polypeptide (757–1035) that covers both MHC class I-restricted (SIINFEKL) and MHC class II-restricted (KISQAVHAAHAEINEAG) OVA epitopes. The two epitopes are underlined. B. Protein sequence of OVA showing both MHC class I-restricted (SIINFEKL) and MHC class II-restricted (KISQAVHAAHAEINEAG) OVA epitopes (underlined). (TIF) [file pone.0145833.s001.tif]

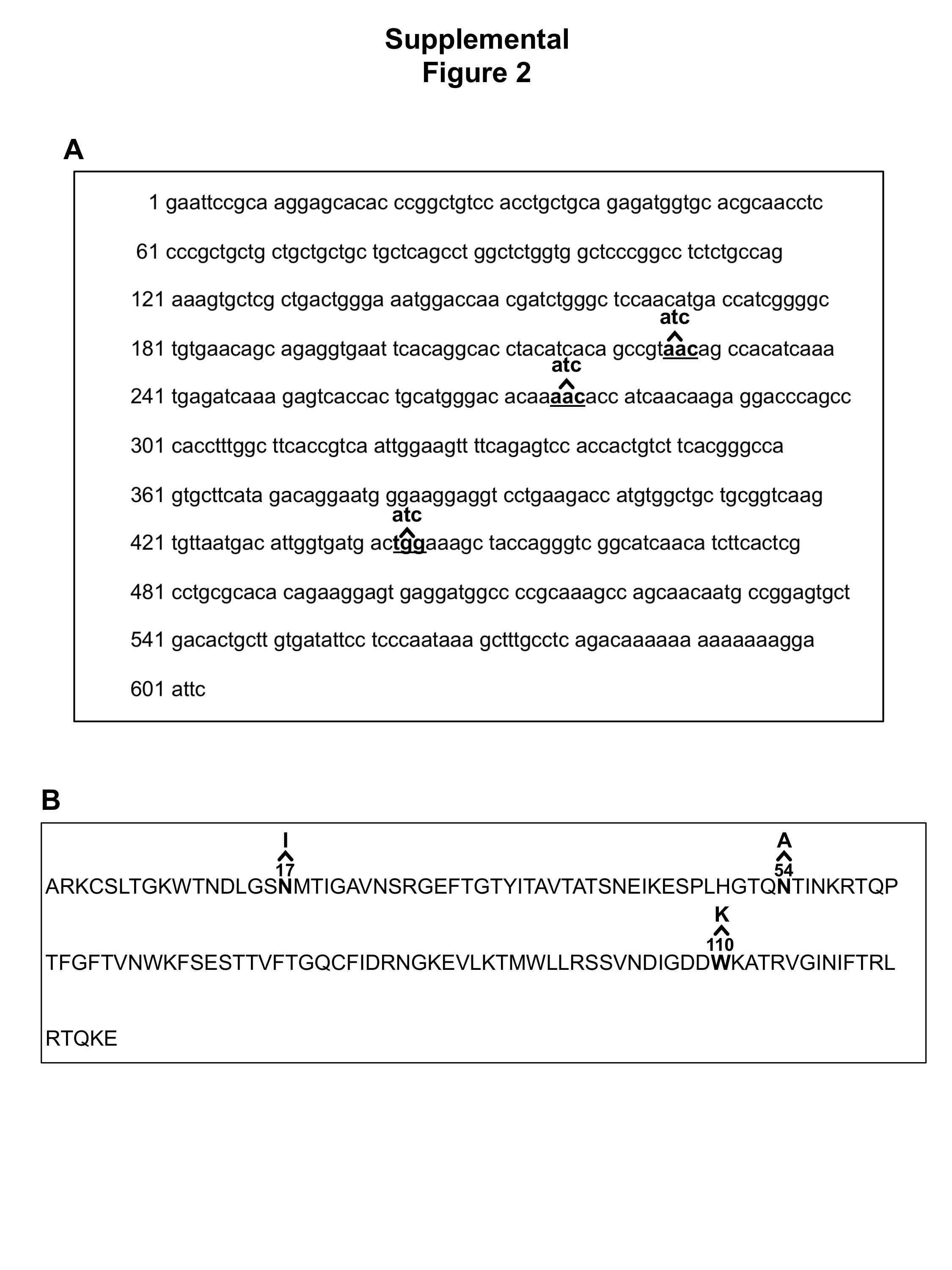

Supplement: S2 Fig — A. DNA sequence showing the three mutations (N17I, N54A & W110K) introduced in wild-type avidin to obtain a monomeric avidin. B. Protein sequence of triple mutant avidin. (TIF) [file pone.0145833.s002.tif]

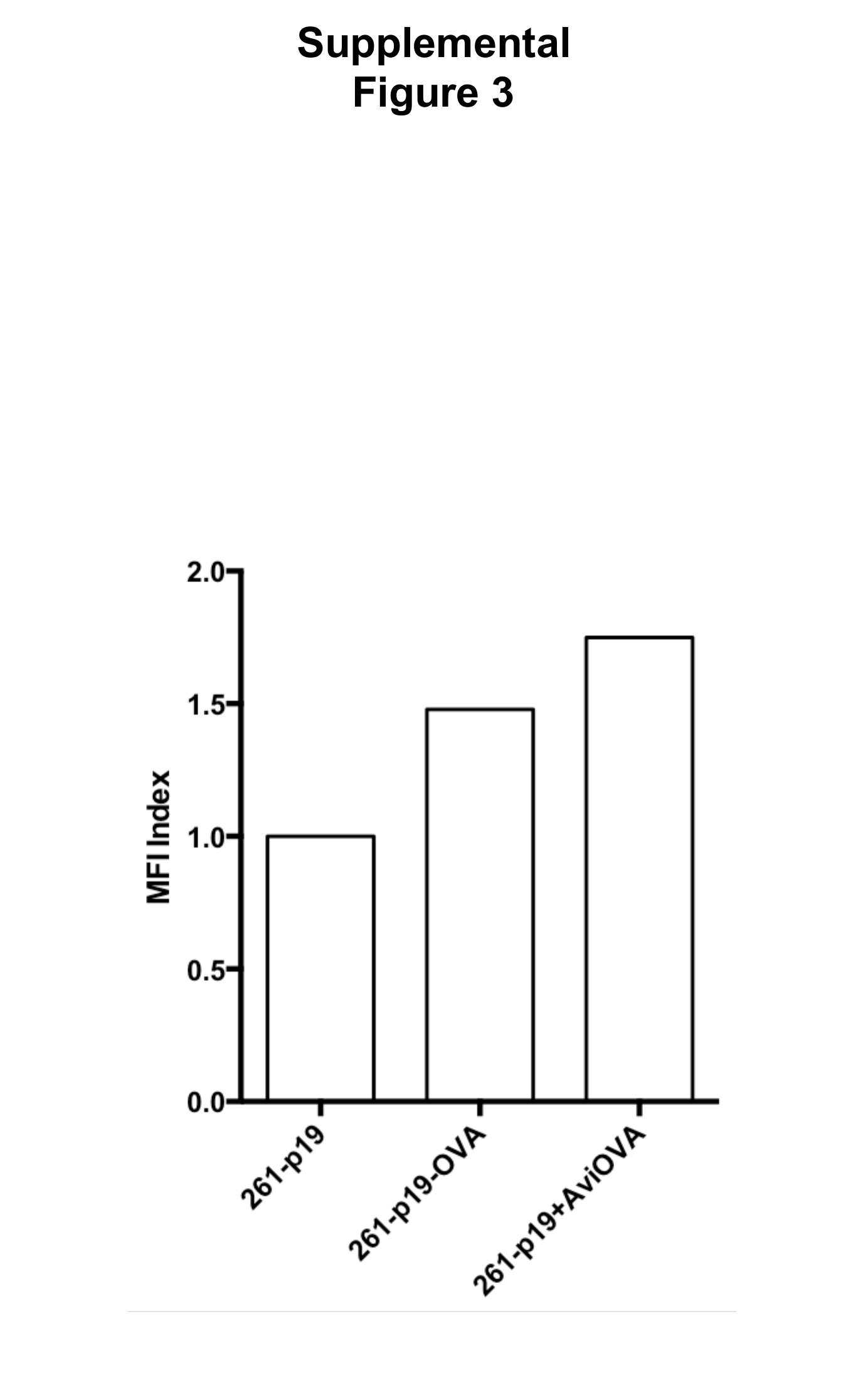

Supplement: S3 Fig — BCG 261-p19-OVA and BCG 261-p19 decorated with Avi-OVA were fixed and stained with mouse anti-OVA Ab and the level of OVA on cell surface was revealed with FITC-anti-mouse IgG. Samples were then analyzed by FACS. Results are presented as MFI indexes, which correspond to the Ratios: MFIs deducted from OVA expressing BCG 261-p19/ MFI corresponding to control BCG-p19 alone. (TIF) [file pone.0145833.s003.tif]

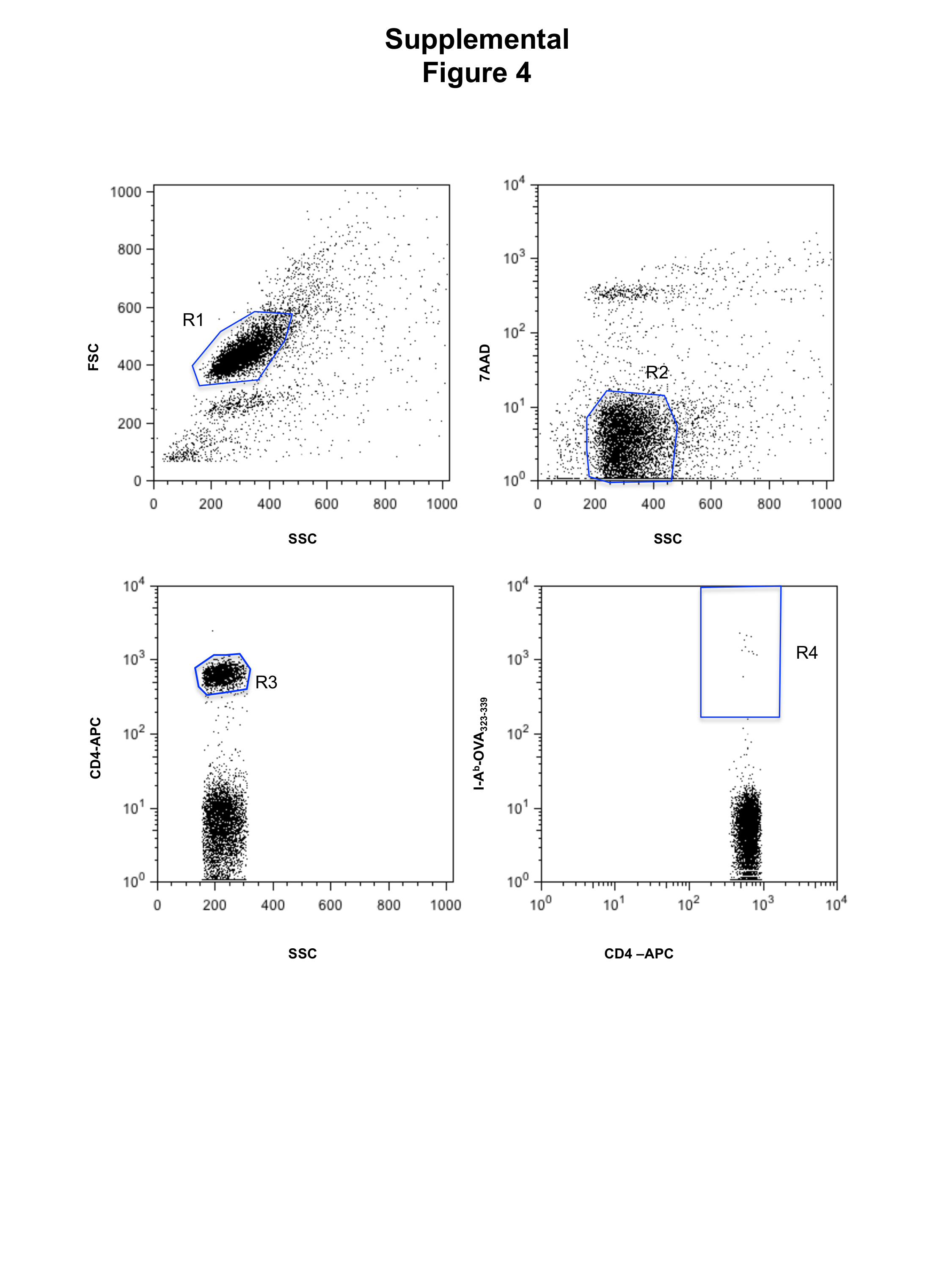

Supplement: S4 Fig — Splenocytes acquired on FACSCalibur flow cytometer were gated on a SSC/FSC (region R1). Live cell (7-AAD negative) were gated in region R2. Total CD4+ (or CD8+) T cells were gated in region R3 to determine frequencies of tetramer positive events (R4). (TIF) [file pone.0145833.s004.tif]
